# Enhancing Arteriovenous (AV) Fistula Banding Accuracy With Intraoperative Ultrasound: A Case Report

**DOI:** 10.7759/cureus.90529

**Published:** 2025-08-19

**Authors:** Adel Hanandeh, Victor Cabrera, Fisher Frederick, Gerard De Castro

**Affiliations:** 1 Vascular Surgery, Univeristy of Central Florida, Orlando, USA; 2 General Surgery, Osceola Regional Medical Center, Orlando, USA; 3 Vascular Surgery, University of Central Florida College of Medicine, Orlando, USA; 4 Vascular Surgery, University of Central Florida College of Medicine, Orlando , USA

**Keywords:** arteriovenous fistula [avf], avf banding, brachiocephalic fistula, flow reduction, hemodialysis access, hemodialysis vascular access monitoring, high-output avf, intraoperative duplex ultrasound, real-time flow assessment

## Abstract

High-output arteriovenous fistulas (AVFs) pose a risk for volume overload, venous hypertension, and right heart strain in hemodialysis patients. We present a case of a 55-year-old male with a symptomatic high-flow brachiocephalic AVF, successfully managed with intraoperative duplex ultrasound-guided banding. Real-time flow measurements enabled precise titration of constriction, reducing flow from 7,891 to 2,000 mL/minute. Postoperative recovery was uneventful, with symptom resolution and preserved access patency. This case demonstrates the clinical utility of intraoperative duplex ultrasound in enhancing surgical accuracy and optimizing outcomes during AVF revision. Broader adoption may improve safety and standardization in managing high-output dialysis access.

## Introduction

Arteriovenous fistulas (AVFs) remain the preferred modality for hemodialysis access due to their superior long-term patency, reduced infection risk, and lower thrombosis rates compared to prosthetic grafts or tunneled dialysis catheters [1,2]. However, managing AVFs with excessively high flow presents a significant clinical challenge. High-output AVFs, typically defined as those with flow rates exceeding 1,500-2,000 mL/minute, can lead to complications such as venous hypertension, aneurysmal dilation, and high-output cardiac failure [1,2].

According to the National Kidney Foundation’s Kidney Disease Outcomes Quality Initiative (NKF-KDOQI), a minimum flow of 600 mL/minute is required for mature AVFs to ensure adequate dialysis delivery. Conversely, flow rates below 400-500 mL/minute may indicate underlying stenosis, while flows exceeding 2,000 mL/minute can contribute to volume overload, right heart strain, and adverse cardiac remodeling [1-3].

AVF banding is a well-established surgical technique for reducing access flow in high-output fistulas [4-6]. However, conventional approaches often rely on subjective intraoperative assessments such as visual inspection or tactile feedback, which may be unreliable and operator dependent [5-8]. This variability can lead to overcorrection, increasing the risk of thrombosis and access loss, or undercorrection, which may leave the patient symptomatic and at continued cardiovascular risk [6].

To mitigate these issues, intraoperative duplex ultrasound has emerged as a valuable adjunct, offering real-time, quantitative measurement of access flow (Qa) [3-5]. This enables titrated banding to physiologic targets while preserving access patency, thereby enhancing both surgical accuracy and clinical outcomes [4,6,7].

A growing body of evidence supports the role of intraoperative duplex ultrasound in vascular access procedures. Malik et al. highlighted its utility in diagnosing stenosis and guiding operative planning [4]. de Castro-Santos et al. demonstrated that intraoperative measurements of peak systolic velocity and Qa can predict early AVF patency [3]. Turner et al. reported improved long-term outcomes using ultrasound-guided AVF banding [5]. These findings advocate for a shift toward physiologically guided access management using real-time hemodynamic data [3-6].

Effective implementation of this approach requires access to high-quality imaging equipment and operator proficiency in vascular ultrasound techniques [4]. Competence in probe positioning, Doppler angle correction, and volume flow calculations is critical for obtaining reproducible results. As such, increased training and comfort with intraoperative ultrasound are likely to be important in standardizing AVF interventions [4,6,7].

This case report describes the use of intraoperative duplex ultrasound to guide surgical banding of a high-output brachiocephalic AVF in a 55-year-old male, achieving controlled flow reduction, symptom resolution, and preserved access function.

## Case presentation

A 55-year-old male with end-stage renal disease (ESRD) on chronic hemodialysis via a left brachiocephalic AVF for the past two years presented with progressive left upper extremity swelling and exertional dyspnea. He had previously undergone aneurysmorrhaphy of the AVF for significant venous aneurysmal dilation. Despite that revision, the patient continued to exhibit clinical signs suggestive of high-output cardiac failure.

**Figure 1:**
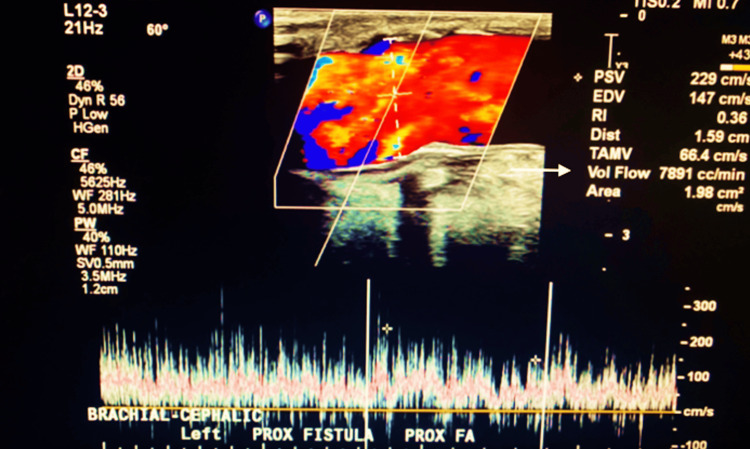
Preoperative duplex ultrasound consistent with a high-output AVF. AVF, arteriovenous fistulas

Physical examination revealed visibly dilated superficial veins over the left upper extremity and chest wall, along with a prominent hyperdynamic thrill over the AVF. Cardiovascular evaluation demonstrated a right ventricular heave and bilateral lower extremity edema. Transthoracic echocardiography demonstrated right ventricular dilation and elevated pulmonary artery pressures, consistent with early right heart strain. (Transthoracic echocardiogram findings were not archived in image format; however, narrative interpretation confirmed right ventricular dilation and elevated pulmonary artery pressures.) Duplex ultrasound of the AVF revealed a high-volume flow of 7,891 mL/minute, confirming the diagnosis of a high-output AVF (Figure 1). The access remained patent, with no evidence of stenosis or thrombosis.

Given the excessive AVF flow and associated cardiopulmonary compromise, a multidisciplinary evaluation was conducted to determine appropriate flow-reduction strategies. Potential options included revision using distal inflow (RUDI), plication, endovascular balloon-assisted banding, and open surgical banding. The decision was made to proceed with open surgical banding under intraoperative duplex ultrasound guidance to allow precise real-time flow assessment and titration.

**Figure 2:**
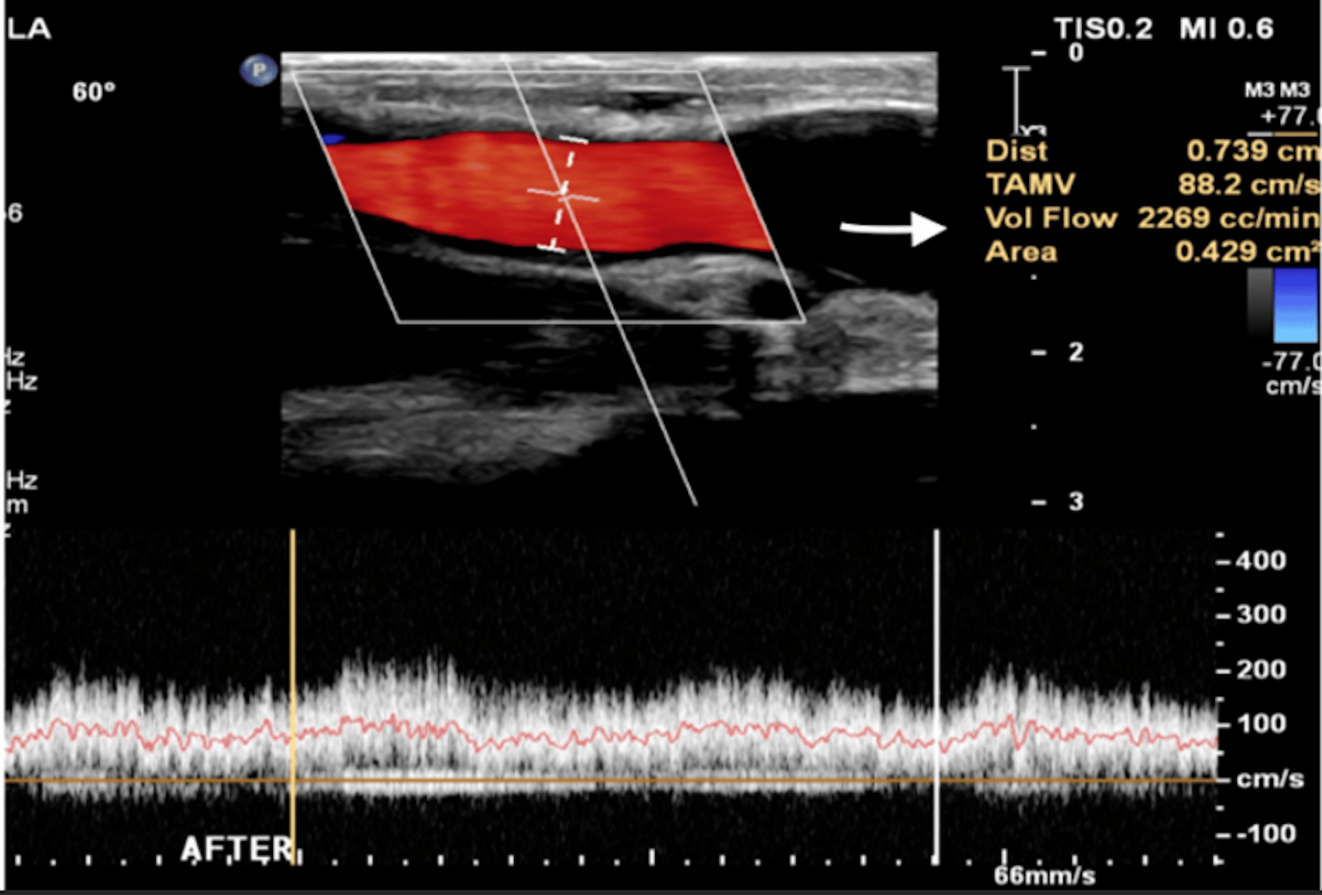
Intraoperative duplex ultrasound illustrating adequate flow volume after banding.

The procedure was performed under regional anesthesia. A longitudinal incision was made over the left upper arm to expose the brachiocephalic AVF. An 8 mm expanded polytetrafluoroethylene (ePTFE) graft was fashioned into a constricting band. Intraoperative duplex ultrasound was used continuously to measure real-time flow distal to the banding site. The band was incrementally tightened, and after each adjustment, flow was reassessed using Doppler ultrasound, targeting a physiologic range between 1,000 and 2,000 mL/minute. Final intraoperative measurements confirmed successful flow reduction from 8,000 mL/minute to approximately 2,000 mL/minute (Figure 2).

**Figure 3:**
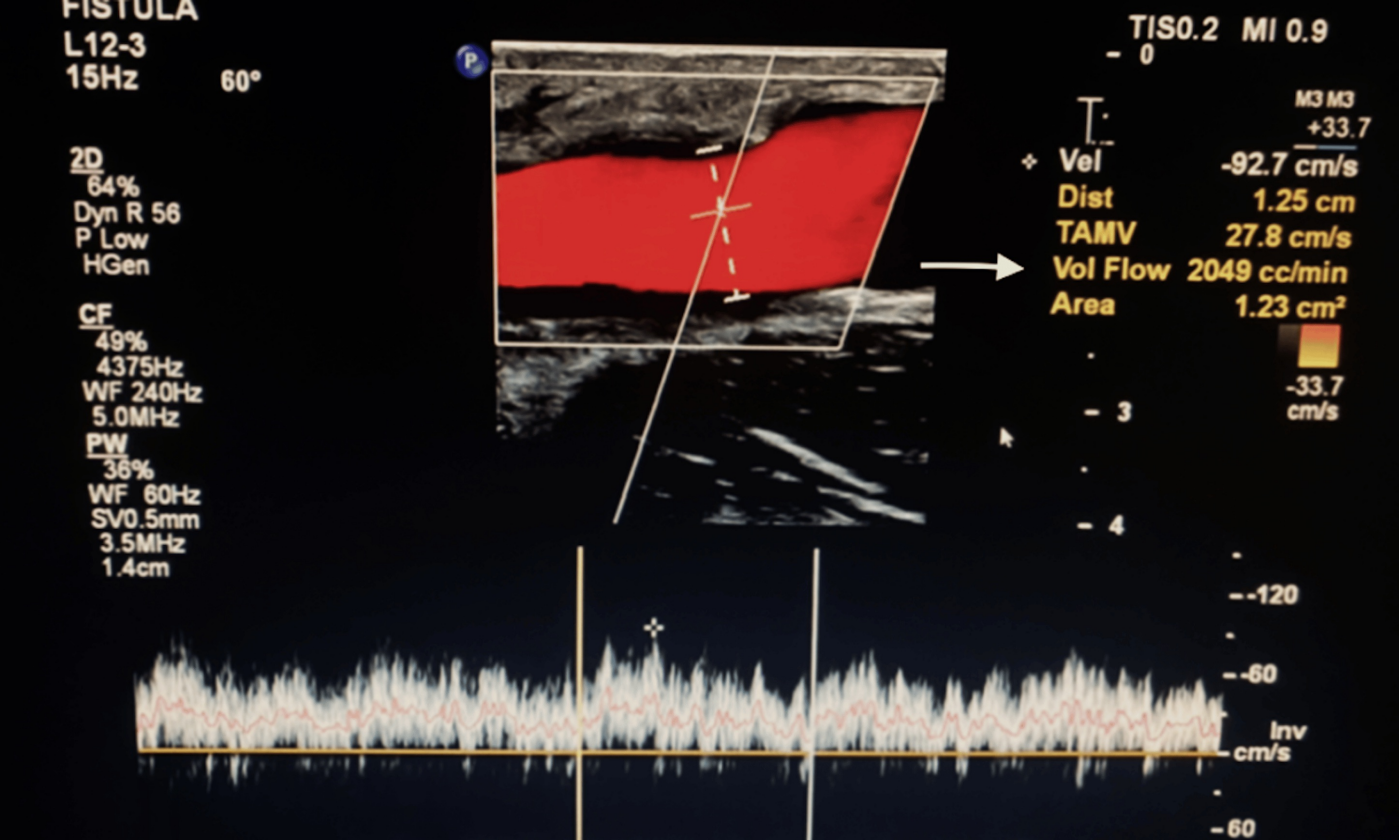
Follow-up duplex ultrasound four weeks after surgery illustrating a consistent reduction in flow volume.

The patient tolerated the procedure without complications. At the four-week postoperative follow-up, duplex ultrasound demonstrated stable AVF flow at 2,049 mL/minute (Figure 3). The fistula remained patent, with no evidence of thrombosis or outflow stenosis. Clinically, the patient reported significant improvement in symptoms, including resolution of upper extremity edema, reduced dyspnea, and improved exercise tolerance.

**Figure 4:**
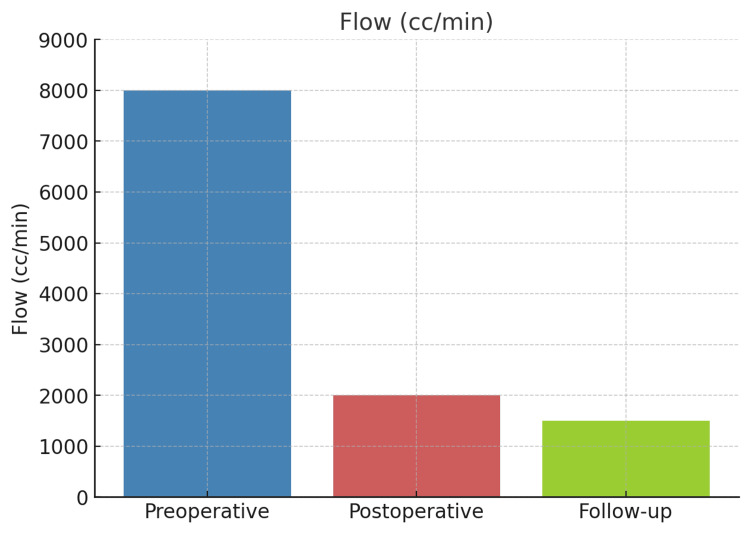
This figure delineates the trend in flow volume from the preoperative period through postoperative follow-up.

This case highlights the utility of intraoperative duplex ultrasound in facilitating physiologic flow reduction from 7,891 mL/minute to approximately 2,000 mL/minute, resulting in both symptomatic relief and preservation of fistula function (Figure 4). The use of real-time ultrasound enhances the precision and safety of AVF revision procedures and may contribute to improved clinical outcomes in patients with high-output vascular access.

## Discussion

High-output AVFs are a recognized complication of long-term hemodialysis access, particularly with brachiocephalic and upper-arm configurations. Excessive arterial shunting into the venous system can result in upper extremity edema, right heart strain, and ultimately congestive heart failure. Flow rates above 1,500-2,000 mL/minute are frequently associated with these complications, and if left untreated, high-output states may lead to irreversible cardiac remodeling [1,2].

Several surgical strategies have been developed to reduce AVF flow, including open banding, plication, revision using distal inflow (RUDI), and balloon-assisted endovascular techniques (Table 1) [6,8]. However, these interventions often rely on subjective intraoperative cues such as palpable thrill or audible Doppler signals, which can be inconsistent and operator dependent [8,9]. This limitation has fueled interest in more objective, quantitative methods of intraoperative flow assessment, particularly duplex ultrasound [3-5].

**Table 1:** Comparison of AVF banding techniques. Adapted from published literature [3,5-9]. AVF, arteriovenous fistula; PTFE, polytetrafluoroethylene

Method	Guidance	Key tool used	Flow control	Risk of over-banding
Open surgical banding	None	Suture/PTFE	Empirical	High
Balloon-assisted banding	Visual only	Compliant balloon	Diameter only	Moderate
Ultrasound-guided banding	Doppler	Ultrasound + band	Yes	Low
Precision flow restrictor	Pre-measured	Graft or cuff	Yes	Low
Hybrid plication + balloon	Visual + tactile	Balloon + plication	Partial	Moderate
Endovascular flow-controlled	Angiographic	Balloon + wire	Partial	Variable

Intraoperative duplex ultrasound enables real-time measurement of volume flow, allowing for titrated adjustments to banding during surgery. In the present case, this technique facilitated precise reduction of AVF flow from 7,891 mL/minute to approximately 2,000 mL/minute, alleviating symptoms of cardiac strain while preserving fistula patency.

The value of intraoperative flow assessment is supported by multiple studies. Turner et al. demonstrated that real-time duplex monitoring significantly reduced complications and improved long-term outcomes over nearly two decades of experience with AVF banding [5]. Intraoperative ultrasound altered surgical plans and improved the accuracy of access creation [7]. Malik et al., in a systematic review, emphasized the broader applicability of duplex ultrasound across the vascular access continuum from AVF creation to revision [4]. de Castro-Santos et al. highlighted the prognostic utility of intraoperative hemodynamic data in predicting early patency [3].

Compared to conventional Doppler, which is highly operator-dependent, duplex ultrasound offers a standardized, quantifiable method of assessing access flow intraoperatively [3,4]. Multiple banding methods have been described, with traditional open surgical banding involving empirical tightening of a nonabsorbable suture or PTFE strip around the vein, often without quantitative guidance [6]. Balloon-assisted techniques, such as the Miller procedure, utilize a compliant intraluminal balloon to guide external constriction, but this method is based on diameter control rather than direct flow measurement [8].

In contrast, ultrasound-guided banding enables direct titration based on real-time volumetric flow calculations [3-5]. Target post-banding flows generally aim for 600-800 mL/minute to ensure adequate dialysis while minimizing the risk of access thrombosis or steal syndrome [1,4,6].

Physiologically guided interventions, particularly when combined with duplex ultrasound and performed by experienced operators, may help reduce complications and the need for reintervention while extending access longevity [3-6]. The broader interventional nephrology literature supports the integration of physiologic feedback into procedural decision-making to improve patient safety and clinical outcomes [3,4].

## Conclusions

This case underscores the critical role of intraoperative duplex ultrasound in optimizing surgical outcomes during revision of high-output arteriovenous fistulas. By providing a real-time, objective assessment of access flow, intraoperative ultrasound enables precise titration of banding to physiologic targets, thereby minimizing the risks of both over- and under-correction. In this patient, guided banding achieved controlled flow reduction from 7,891 mL/minute to approximately 2,000 mL/minute, resulting in significant symptomatic improvement and preservation of fistula function.

As vascular access management continues to move toward individualized, physiology-based strategies, intraoperative duplex ultrasound emerges as a reproducible and efficient tool for enhancing surgical precision and decision-making. Broader adoption of this technique may help reduce postoperative complications, lower reintervention rates, and improve long-term AVF durability in complex or high-risk patients. Future prospective studies are warranted to validate its efficacy across diverse populations and establish standardized intraoperative flow targets and protocols.
